# All-trans retinoic acid stimulates the secretion of TGF-β2 via the phospholipase C but not the adenylyl cyclase signaling pathway in retinal pigment epithelium cells

**DOI:** 10.1186/s12886-018-1017-6

**Published:** 2019-01-18

**Authors:** Daren Zhang, Zhihong Deng, Jia Tan, Shuirong Liu, Shuyu Hu, Hui Tao, Renhong Tang

**Affiliations:** 1 Department of Ophthalmology, The Third Xiangya Hospital, Central South, Changsha, Hunan China; 2 Department of Ophthalmology, Xiangya Hospital, Central South University, Changsha, Hunan China

**Keywords:** Transforming growth factor-β_2_, U73122, SQ22536, Retinal pigment epithelium cells, All-trans retinoic acid

## Abstract

**Background:**

By investigating that (i) all-trans retinoic acid (ATRA) affects human retinal pigment epithelium (RPE) in expressing and secreting transforming growth factor (TGF)-β_2_ and (ii) U73122 (phospholipase C inhibitor) and SQ22536 (adenylyl cyclase inhibitor) regulate the ATRA-induced secretion of TGF-β_2_ in human RPE, we sought to interpret the signaling pathway of ATRA in promoting the development of myopia.

**Methods:**

The RPE cell line (D407) was treated with (i) ATRA (10 μM), (ii) U73122 (5–40 μM) and ATRA (10 μM), or (iii) SQ22536 (5–40 μM) and ATRA (10 μM). The control group was no-treated. After stimulated at 2, 4, 8, 16, 24, and 48 h, The expression and secretion of TGF-β_2_ was detected.

**Results:**

TGF-β2 in the cytoplasm was time-dependent increased by ATRA (*p* < 0.001). A time-dependent increase in the TGF-β2 protein of the supernatant was induced by ATRA (*p* < 0.001). U73122 (in the range of 5 to 40 μM) could suppress the secretion of TGF-β2 induced by ATRA (*p* < 0.001), and 40 μM U73122 could completely inhibit the up-regulated effect of 10 μM ATRA. However, SQ22536 (in the range of 5 to 40 μM) had no impact on the secretion of TGF-β_2_ induced by ATRA (*p* > 0.05).

**Conclusions:**

In RPE cells, ATRA stimulates the secretion of TGF-β_2_ via the phospholipase C signaling pathway but not the adenylyl cyclase signaling pathway. U73122 may inhibit the promotion of ATRA in the development of myopia.

## Background

Retinoic acid (RA) is a biological activity meditator with many functions including proliferation, cell differentiation and apoptosis [1, 2]. It has been reported that RA may be a retinal signal involved in inhibiting the remodeling of scleral extracellular matrix, resulting in slower ocular growth. Yuko Sekoa found that retinal RA increased within 5 days after form-deprivation in the chicken eyes. McFadden SA found that retinal RA accelerates the elongation of the eyes and increases in the myopic eyes of guinea pig. And conversely, it becomes lower in the inhibited elongation eyes [3–8]. Previous studies have reported that all-trans retinoic acid (ATRA) can not only up-regulate the expression of tight junction-associated protein in the retinal pigment epithelium (RPE)-choroid complex and also promotes the epithelial barrier function of the RPE monolayer during the development of myopia [9, 10]. All of these results indicate that RPE plays an important role in the retinal RA signal controlling the scleral remodeling of myopia. It was reported that several cytokines synthesized and secreted by RPE cells have been involved in the progression of myopia [11–13]. Of these factors, it has been proved that transforming growth factor (TGF)-β2 affects the production of collagen and proliferation of scleral fibroblast cells and control the development of myopia [14]. TGFβ is both a critical mediator of collagen, MMP-2, and proteoglycan production in the extracellular matrix. It implies that TGFβ may have potential influence in controlling scleral remodeling, which induces myopia development [15–20]. According to these studies, we infer that retinal RA regulates the TGF-β2 level of RPE in the development of myopia, which controls the scleral remodeling. However, the mechanism by which ATRA could be transported from the choroid to the sclera has not been clarified. In the present study, we sought the effect of ATRA on the expression and secretion of human RPE in vitro and inhibited the signaling pathways of RPE with U73122 (phospholipase C and A2 inhibitor) and SQ22536 (adenylyl cyclase inhibitor) in order to survey which signaling pathway inhibitor can suppress the changes of TGF-β2 induced by ATRA in RPE.

## Methods

### Materials and reagents

In this study, human RPE cell line (D407) was purchased from the Retinal Cell Biology Laboratory, University of South Carolina (Columbia, South Carolina, USA). All-trans retinoic acid (ATRA), U73122, SQ22536 and DMSO were purchased from Sigma-Aldrich (Poole, UK). The human TGF-β_2_ ELISA kit was purchased from Bender MedSystems (Austria). Antibodies of TGF-β_2_ and β-actin were purchased from Santa Cruz (Santa Cruz, CA, USA). Complete protease inhibitor cocktail tablets were obtained from Roche Diagnostics (Roche, Switzerland). The protein marker was purchased from NEB (New England Biolabs, China), and the ECL western blot kit was purchased from Pierce (Lockford, IL, USA). All-trans retinoic acid (ATRA), U73122 and SQ22536 were dissolved in DMSO for subsequent coculture with cells.

### Cell culture and treatment

D407 cells, cultured as previously described [21]. There were 4 groups, Group A: Cells were treated with 10 μM ATRA for 2, 4, 8, 16, 24 and 48 h; Group B: Cells were treated with U73122 (B_1_, 5 μM; B_2_, 10 μM; B_3_, 20 μM; B_4_, 40 μM) for 30 min, then treated with 10 μM ATRA for 24 h; Group C: Cells were treated with SQ22536 (C_1_, 5 μM; C_2_, 10 μM; C_3_, 20 μM; C_4_, 40 μM) for 30 min, then treated with 10 μM ATRA for 24 h; Group D: Cells treated only with equivalent volume of DMSO served as blank controls.

### Western blot analysis and ELISA

Western blot assay and ELISA were performed according to our previous study [21].

### Data and statistical analysis

SPSS software 18.0 (IBM Corp, NY, USA) was used to perform the statistical analysis in the present study. Data are presented as the mean ± SD. Student’s *t*-test (SNK) and One-way ANOVA were used to determine the level of significance; *p < 0.05* was defined as statistically significant. All in vitro cell experiments were repeated three times.

## Results

### ATRA stimulates the expression and secretion of TGF-β_2_ in D407 cells

**Fig. 1:**
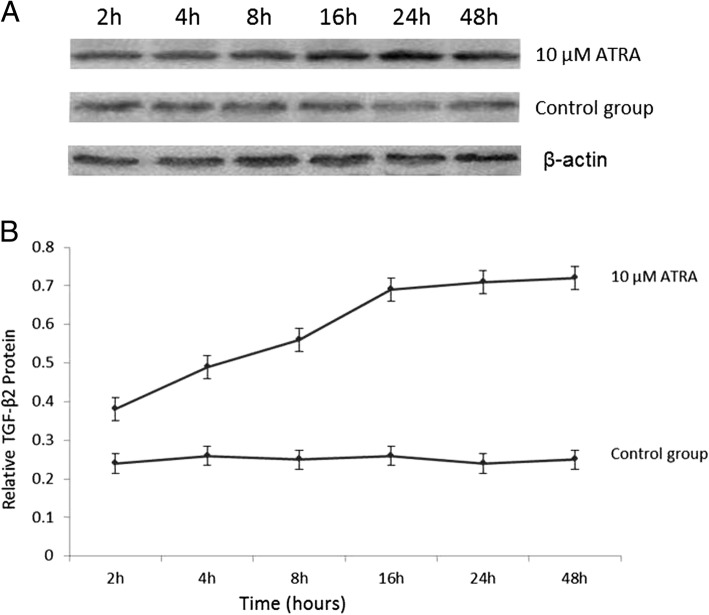
ATRA stimulated the expression of TGF-β_2_ mRNA in D407 cells. D407 cells were treated with 10 μM ATRA for 2, 4, 8, 16, 24, and 48 h, and the expression of TGF-β_2_ protein was detected by western blot analysis**. a** The electrophoretogram of TGF-β_2_ protein in the 10 μM ATRA-treated and control groups for 2, 4, 8, 16, 24, and 48 h. It was found that 10 μM ATRA stimulated the expression of TGF-β_2_ protein in a time-dependent manner. **b** After treatment with ATRA for 2 h, the level of TGF-β_2_ protein in D407 cells was increased significantly compared with that of the control group (*p* < 0.001) and peaked at 16 h. However, there were no statistically significant differences in the level of TGF-β_2_ protein at 16 h, 24 h and 48 h (*p* > 0.05; *n* = 6 per treatment)

**Fig. 2:**
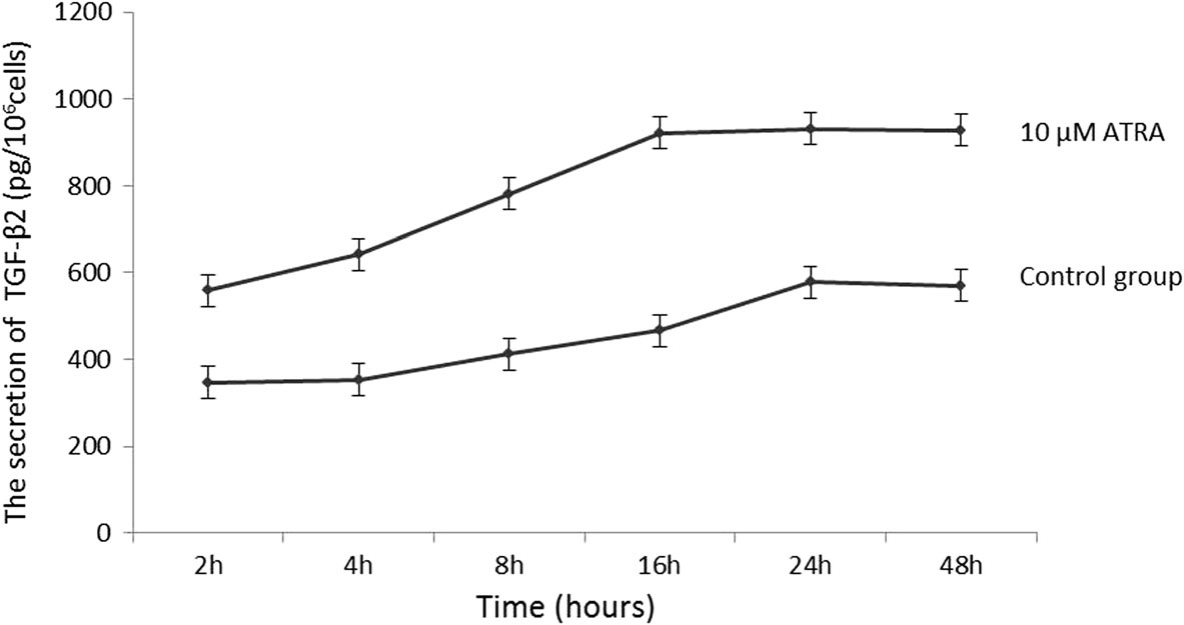
Treatment with 10 μM ATRA stimulated the secretion of TGF-β_2_ protein in the supernatants of D407 cells. TGF-β_2_ protein in the conditioned media was measured by ELISA and normalized to cell counts (1 × 10^6^). The concentration of secreted TGF-β_2_ in the control group increased at 8 h and peaked at 24 h, and there was no statistically significant difference between 24 h and 48 h. After treatment with 10 μM ATRA for 2 h, the concentration of secreted TGF-β_2_ of the ATRA-treat group increased (*p* < 0.001) and peaked at 16 h. However, there was no statistically significant difference the concentrations of secreted TGF-β_2_ in the 10 μM ATRA-treated group at 16 h, 24 h and 48 h (*p* > 0.05; *n* = 3 per treatment). The effects of U73122 on the ATRA-induced secretion of TGF-β2 in D407 Cells . Cells were pretreated with U73122 (5 μM, 10 μM, 20 μM and 40 μM) for 30 min, followed by exposure to ATRA (10 μM) for 24 h. After treatment with 5–40 40 μM U73122 + 10 μM ATRA, the concentrations of secreted TGF-β2 in the supernatants were significantly lower than those of the ATRA-treated group (*p* < 0.01). The concentration of secreted TGF-β2 decreased with the increase of U73122. When the concentration of U73122 reached 40 μM, the concentration of secreted TGF-β2 was not significantly different from that of the control group (*p* > 0.05) (Fig. 3). The results indicated that the secretion of TGF-β_2_ induced by ATRA is inhibited by U73122 (5–40 μM) in D407 cells. This suppressive effect of U73122 was enhanced with increasing concentrations, and the effect of 10 μM ATRA was completely inhibited by 40 μM U73122

To identify whether ATRA can stimulate D407 cells in expressing and secreting TGF-β_2_, we examined the concentration of TGF-β_2_ in the cytoplasm, and the level of TGF-β2 secreted in the supernatant in the ATRA treatment group and control group at 2, 4, 8, 16, 24 and 48 h. In the control group, the concentration of TGF-β_2_ in the cytoplasm did not change in the whole time period (*p* > 0.05) (Fig. 1). The level of TGF-β_2_ of the control group in the supernatant increased at 8 h and peaked at 24 h (Fig. 2) At each time point, the expression and secretion of TGF-β_2_ in the ATRA treated group were significantly higher than in the control group (*p* < 0.001). The TGF-β_2_ level in the cytoplasm and supernatant increased at 2 h and peaked at 16 h. However, there were no significant differences between the concentrations of TGF-β_2_ and the levels of secreted TGF-β_2_ at 16 h, 24 h and 48 h. (*p* > 0.05) (Figs. 1, 2). These results proved that ATRA could induce the significant up-regulation of TGF-β_2_ in both cytoplasm and supernatant in a time-dependent manner.

### The effects of U73122 on the ATRA-induced secretion of TGF-β2 in D407 cells

**Fig. 3:**
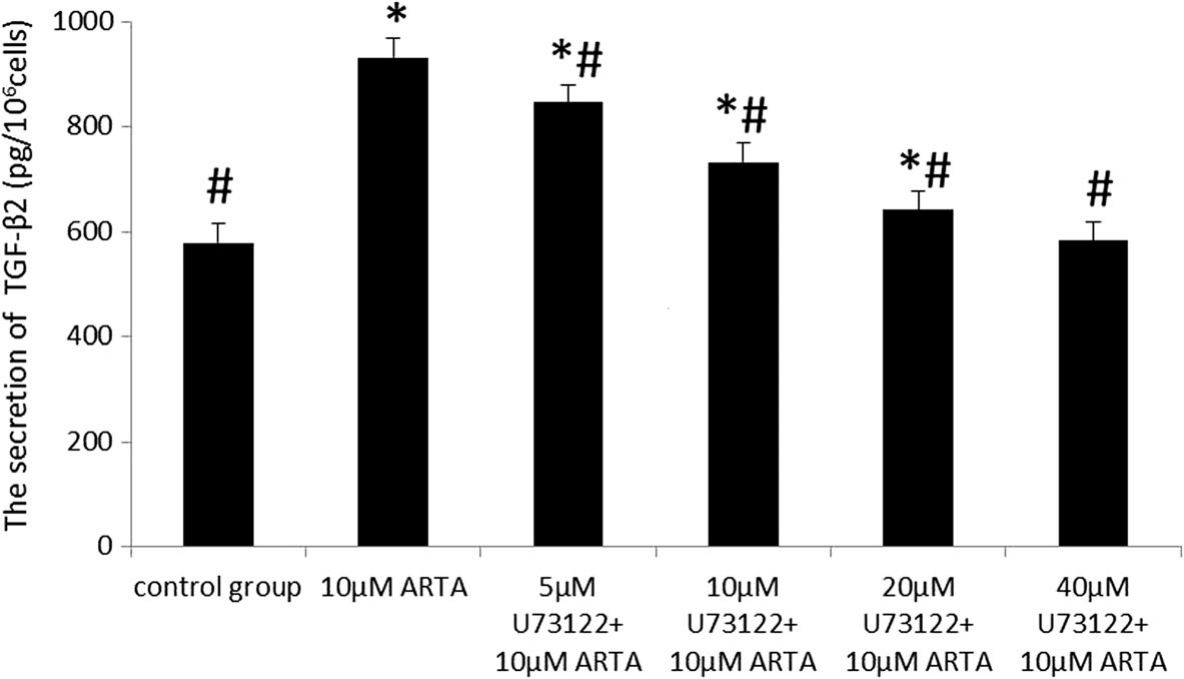
U73122 suppressed the quantity of TGF-β_2_ protein induced by ATRA in the supernatants of D407 cells. TGF-β_2_ protein levels in conditioned media were measured by ELISA and normalized to cell counts (1 × 10^6^). The secretion of TGF-β_2_ induced by ATRA was inhibited by U73122 (5–40 μM) in D407 cells. The suppressive effect of U73122 was enhanced with increasing concentrations of U73122, and the effect of 10 μM ATRA was completely inhibited by 40 μM U73122. Data are represented as the mean ± SEM. Statistical comparisons were performed by one-way ANOVA. *Significance versus control group (*p* < 0.001; *n* = 3 per treatment). ^#^Significance versus ATRA-treated group (10 μM) (*p* < 0.001; n = 3 per treatment). The effects of SQ22536 on the ATRA-induced secretion of TGF-β2 in D407 Cells. Cells were pretreated with SQ22536 (5 μM, 10 μM, 20 μM and 40 μM) for 30 min, followed by exposure to ATRA (10 μM) for 16 h. There was no significant difference between the secretion of TGF-β_2_ in the 5–40 μM SQ22536 + ATRA-treated group and ATRA-treated group (*p* > 0.05) (Fig. 4). These results indicate that SQ22536 (5–40 μM) had no suppressive effects on the secretion of TGF-β_2_ induced by ATRA in D407 cells

Cells were pretreated with U73122 (5 μM, 10 μM, 20 μM and 40 μM) for 30 min, then exposure to ATRA (10 μM) for 24 h. After treatment with (5-40 μM) U73122 + 10 μM ATRA, the concentrations of secreted TGF-β2 in the supernatants were significantly lower than those of the ATRA-treated group (*p* < 0.01). The concentration of secreted TGF-β2 decreased with the increase of U73122. When the concentration of U73122 reached 40 μM, the concentration of secreted TGF-β2 was not significantly different from that of the control group (*p* > 0.05) (Fig. 3). The results indicated that the secretion of TGF-β_2_ induced by ATRA is inhibited by U73122 (5–40 μM) in D407 cells. This suppressive effect of U73122 was enhanced with increasing concentrations, and the effect of 10 μM ATRA was completely inhibited by 40 μM U73122.

### The effects of SQ22536 on the ATRA-induced secretion of TGF-β2 in D407 cells

**Fig. 4:**
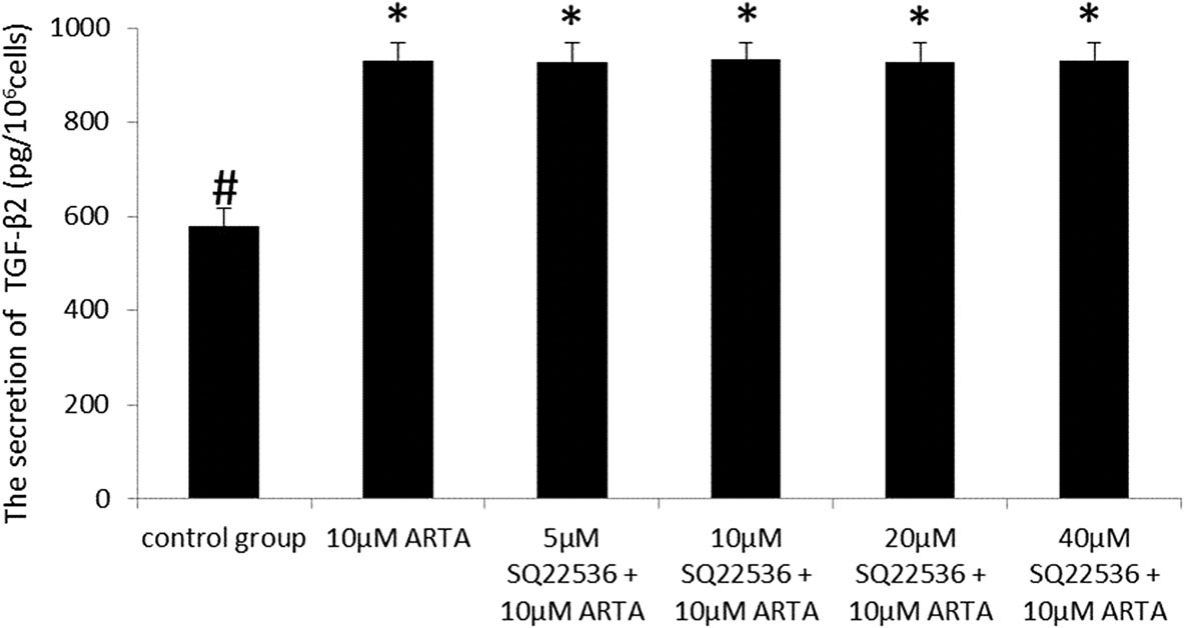
SQ22536 did not suppress the quantity of TGF-β_2_ protein induced by ATRA in the supernatants of D407 cells. TGF-β_2_ protein levels in conditioned media were measured by ELISA and normalized to cell counts (1 × 10^6^). There was no significant difference in the secretion of TGF-β_2_ of the ATRA-treated group and SQ22536 (5–40 μM) + ATRA-treated group (*p* > 0.05). Data are represented as the mean ± SEM. Statistical comparisons were performed with one-way ANOVA. *Significance versus control group (*p* < 0.001; *n* = 3 per treatment). ^#^Significance versus ATRA-treat group (10 μM) (*p* < 0.001; *n* = 3 per treatment)

Cells were pretreated with SQ22536 (5 μM, 10 μM, 20 μM and 40 μM) for 30 min, followed by exposure to ATRA (10 μM) for 16 h. There was no significant difference between the secretion of TGF-β_2_ in the (5–40 μM) SQ22536 + ATRA-treated group and ATRA-treated group (*p* > 0.05) (Fig. 4). These results indicate that SQ22536 (5-40 μM) had no suppressive effects on the secretion of TGF-β_2_ induced by ATRA in D407 cells.

## Discussion

The metabolism of Vitamin A and its derivative retinoid happen in the retina. As a product of Vitamin A metabolism, RA is a critical factor of maintaining the normal functions of the eye. It has been reported that the structure and functions of RPE cells is regulated by RA [22]. RA causes growth arrest of RPE cells and induces hexagonal changes in their shapes, which plays a crucial role in maintaining the RPE’s monolayer structure [23]. In addition, RA was reported to up-regulate TSP-1 and enhance the concentration of TGF-β1 in RPE cells [24]. In our research, we attempted to investigate the effect of RA on TGF-β2, which is closely related to myopia. We found that ATRA could up-regulate the expression of TGF-β2 in the RPE cells and increase the secretion of TGF-β2 time-dependently. In the mice, guinea pig and mouse, TGF-β2 was proved to be expressed in the retina, RPE-choroid complex and sclera [25, 26]. In a recent study, the TGF-β2 concentration of aqueous in highly myopic patients was significantly up-regulated, and was positively related to axial length of myopic patients.. According to these results, we inferred that increased RA in the retina of patients with myopia might induce the up-regulation of TGF-β2 in the RPE, which leads to the chondrogenic change in the sclera during the development of myopia. Nonetheless, the signaling pathway by which RA regulates TGF-β2 in RPE was not clear until now.

In the RPE, RA is bound to the retinoic X receptor (RXR) and retinoic acid receptor (RAR) to regulate gene transcription. There are two signaling pathways in RPE: (i) phospholipase C (PLC), calcium(Ca^2+^) channels, and protein kinase C (PKC), and (ii) adenylyl cyclase, calcium (Ca^2+^) channels, and cyclic AMP (cAMP)-dependent protein kinase A (PKA) [27]. In our study, we used two inhibitors (U73122 and SQ22536) to inhibit the signaling pathways. As the inhibitor of phospholipase C and A2, U73122 can reduce the free cytosolic Ca2^+^ via inhibiting the hydrolysis of PPI (phosphatidylinositol) to IP3 (inositol triphosphate). It also inhibits the activation of G-protein phospholipase C coupling while remaining unaffected by the production of cAMP [28]. SQ22536 is a cell-permeable adenylyl cyclase inhibitor. SQ22536 reduces the activity of adenylate cyclase, when the concentrations of GTP increases with Mg^++^ and Mn^++^, SQ22536 can inhibit that the activating effect of Mg^++^ and Mn^++^ on adenylate cyclase in the presence of catecholamines, [29]. We found that after treatment with U73122 + ATRA, the concentration of secreted TGF-β2 was significantly lower than that of the ATRA-treated group. When the concentration of U73122 reached to 40 μM, the concentration of secreted TGF-β2 was not significantly different from that of the control group. These results indicated that ATRA stimulated the secretion of TGF-β_2_ via the PKC signaling pathway in RPE. On the other hand, we found that in the SQ22536-treated groups, there was no significant difference between the secretion of TGF-β_2_ in the SQ22536 + ATRA-treated group and the ATRA-treated group, which indicated that the adenylyl cyclase signaling pathway was not involved in the effect of the stimulation of ATRA on the secretion of TGF-β_2_. We concluded that retinal RA might bind to the RA receptors of RPE and activate the PKC signaling pathway, stimulating the secretion of TGF-β_2_ in the RPE and inducing scleral remodeling in the myopia.

In further studies, our group will inject ATRA and U73122 into the sub-retina of mice, observe the effects of the suppression of U73122 on the changes in RPE induced by ATRA, and determine whether the growth of eyeball induced by ATRA can be suppressed by U73122. We will attempt to clarify the mechanism behind ATRA’s control of myopia.

## Conclusions

In RPE cells, ATRA stimulates the secretion of TGF-β_2_ via the phospholipase C signaling pathway but not the adenylyl cyclase signaling pathway. U73122 may inhibit the secretion of TGF-β2 induced by the stimulation of ATRA, which slow down the scleral remodeling in the myopia.

## Abbreviations

ATRA: All-trans retinoic acid
BCA: Bicinchoninic acid
DMEM: Dulbecco’s modified Eagle’s medium
ECL: Enhanced chemiluminescence
ELISA: Enzyme-linked immunosorbent assay
GTP: Guanosine triphosphate
PBS: Phosphate buffer saline
PKA: Protein kinase A
PKC: Protein kinase C
PLC: Phospholipase C
RA: Retinoic acid
RAR: Retinoic acid receptor
RPE: Retinal pigment epithelium
RXR: Retinoic X receptor
TGF: Transforming growth factor
TMB: Tetramethylbenzidine

## References

[1] Durston AJ, Timmermans JP, Hage WJ, Hendriks HF, de Vries NJ, Heideveld M, Nieuwkoop PD (1989). Retinoic acid causes an anteroposterior transformation in the developing central nervous system. Nature.

[2] Osanai M, Petkovich M (2005). Expression of the retinoic acid-metabolizing enzyme CYP26A1 limits programmed cell death. Mol Pharmacol.

[3] Bitzer M, Feldkaemper M, Schaeffel F (2000). Visually induced changes in components of the retinoic acid system in fundal layers of the chick. Exp Eye Res.

[4] Huang J, Qu XM, Chu RY (2011). Expressions of cellular retinoic acid binding proteins I and retinoic acid receptor-beta in the Guinea pig eyes with experimental myopia. Int J Ophthalmol..

[5] McFadden SA, Howlett MH, Mertz JR (2004). Retinoic acid signals the direction of ocular elongation in the Guinea pig eye. Vis Res.

[6] McFadden SA, Howlett MH, Mertz JR, Wallman J (2006). Acute effects of dietary retinoic acid on ocular components in the growing chick. Exp Eye Res.

[7] Seko Y, Shimizu M, Tokoro T (1998). Retinoic acid increases in the retina of the chick with form deprivation myopia. Ophthalmic Res.

[8] Troilo D, Nickla DL, Mertz JR, Summers Rada JA (2006). Change in the synthesis rates of ocular retinoic acid and scleral glycosaminoglycan during experimentally altered eye growth in marmosets. Invest Ophthalmol Vis Sci.

[9] Mao J, Liu S (2014). Regulation of RPE barrier function by all-trans retinoic acid in myopia. Neurosci Lett.

[10] Wang S, Liu S, Mao J, Wen D (2014). Effect of retinoic acid on the tight junctions of the retinal pigment epithelium-choroid complex of Guinea pigs with lens-induced myopia in vivo. Int J Mol Med.

[11] Hollborn M, Enzmann V, Barth W, Wiedemann P, Kohen L (2000). Changes in the mRNA expression of cytokines and chemokines by stimulated RPE cells in vitro. Curr Eye Res.

[12] Kociok N, Heppekausen H, Schraermeyer U, Esser P, Thumann G, Grisanti S, Heimann K (1998). The mRNA expression of cytokines and their receptors in cultured iris pigment epithelial cells: a comparison with retinal pigment epithelial cells. Exp Eye Res.

[13] Rosenthal R, Malek G, Salomon N, Peill-Meininghaus M, Coeppicus L, Wohlleben H, Wimmers S, Bowes RC, Strauss O (2005). The fibroblast growth factor receptors, FGFR-1 and FGFR-2, mediate two independent signalling pathways in human retinal pigment epithelial cells. Biochem Biophys Res Commun.

[14] Nagineni CN, Cherukuri KS, Kutty V, Detrick B, Hooks JJ (2007). Interferon-gamma differentially regulates TGF-beta1 and TGF-beta2 expression in human retinal pigment epithelial cells through JAK-STAT pathway. J Cell Physiol.

[15] Jia Y, Hu DN, Zhou J (2014). Human aqueous humor levels of TGF- beta2: relationship with axial length. Biomed Res Int.

[16] Chen BY, Wang CY, Chen WY, Ma JX (2013). Altered TGF-beta2 and bFGF expression in scleral desmocytes from an experimentally-induced myopia Guinea pig model. Graefes Arch Clin Exp Ophthalmol.

[17] Lin HJ, Wan L, Tsai Y, Liu SC, Chen WC, Tsai SW, Tsai FJ (2009). Sclera-related gene polymorphisms in high myopia. Mol Vis.

[18] Jobling AI, Wan R, Gentle A, Bui BV, McBrien NA (2009). Retinal and choroidal TGF-beta in the tree shrew model of myopia: isoform expression, activation and effects on function. Exp Eye Res.

[19] Jobling AI, Gentle A, Metlapally R, McGowan BJ, McBrien NA (2009). Regulation of scleral cell contraction by transforming growth factor-beta and stress: competing roles in myopic eye growth. J Biol Chem.

[20] Jobling AI, Nguyen M, Gentle A, McBrien NA (2004). Isoform-specific changes in scleral transforming growth factor-beta expression and the regulation of collagen synthesis during myopia progression. J Biol Chem.

[21] Tan J, Deng ZH, Liu SZ, Wang JT, Huang C (2010). TGF-beta(2) in human retinal pigment epithelial cells: expression and secretion regulated by cholinergic signals in vitro. Curr Eye Res.

[22] Campochiaro PA, Hackett SF, Conway BP (1991). Retinoic acid promotes density-dependent growth arrest in human retinal pigment epithelial cells. Invest Ophthalmol Vis Sci.

[23] Uchida H, Hayashi H, Kuroki M, Uno K, Yamada H, Yamashita Y, Tombran-Tink J, Kuroki M, Oshima K (2005). Vitamin a up-regulates the expression of thrombospondin-1 and pigment epithelium-derived factor in retinal pigment epithelial cells. Exp Eye Res.

[24] Uchida H, Kuroki M, Shitama T, Hayashi H, Kuroki M (2008). Activation of TGF-beta1 through up-regulation of TSP-1 by retinoic acid in retinal pigment epithelial cells. Curr Eye Res.

[25] Mao JF, Liu SZ (2013). Mechanism of the DL-alpha-aminoadipic acid inhibitory effect on form-deprived myopia in Guinea pig. Int J Ophthalmol.

[26] Wu PC, Tsai CL, Gordon GM, Jeong S, Itakura T, Patel N, Shi S, Fini ME (2015). Chondrogenesis in scleral stem/progenitor cells and its association with form-deprived myopia in mice. Mol Vis.

[27] Das BC, Thapa P, Karki R, Das S, Mahapatra S, Liu TC, Torregroza I, Wallace DP, Kambhampati S, Van VP, Verma A, Ray SK (2014). Evans T (2014) retinoic acid signaling pathways in development and diseases. Bioorg Med Chem.

[28] Thoreson WB, Ryan JS, Shi C, Kelly ME, Bryson EJ, Toews ML, Ediger TL, Chacko DM (2002). Lysophosphatidic acid receptor signaling in mammalian retinal pigment epithelial cells. Invest Ophthalmol Vis Sci.

[29] Tao Y, Pan M, Liu S, Fang F, Lu R, Lu C, Zheng M, An J, Xu H, Zhao F, Chen JF, Qu J, Zhou X. cAMP level modulates scleral collagen remodeling, a critical step in the development of myopia. PLoS One. 2013. 10.1371/journal.pone.0071441. 10.1371/journal.pone.0071441 PMC3741144 23951163

